# Effect of Acupuncture vs Sham Procedure on Chemotherapy-Induced Peripheral Neuropathy Symptoms: A Randomized Clinical Trial

**DOI:** 10.1001/jamanetworkopen.2020.0681

**Published:** 2020-03-11

**Authors:** Ting Bao, Sujata Patil, Connie Chen, Iris W. Zhi, Qing S. Li, Lauren Piulson, Jun J. Mao

**Affiliations:** 1 Integrative Medicine Service, Memorial Sloan Kettering Cancer Center, New York, New York; 2 Department of Epidemiology and Biostatistics, Memorial Sloan Kettering Cancer Center, New York, New York; 3 Breast Medicine Service, Memorial Sloan Kettering Cancer Center, New York, New York

## Abstract

This randomized clinical trial investigates the effect of acupuncture vs a sham procedure or usual care for chemotherapy-induced peripheral neuropathy symptoms.

## Introduction

Chemotherapy-induced peripheral neuropathy (CIPN) is the most common and debilitating long-term adverse effect of neurotoxic chemotherapy that significantly worsens cancer survivors’ quality of life. Well-tolerated, evidence-based interventions for CIPN are needed.^1^

## Methods

This pilot randomized clinical trial compared the effect of 8 weeks of real acupuncture vs sham acupuncture or usual care to treat CIPN. The trial protocol (Supplement 1) was approved by the institutional review board of Memorial Sloan Kettering Cancer Center and follows the Consolidated Standards of Reporting Trials (CONSORT) reporting guideline (Figure). Written informed consent was obtained from all study participants. Patients with solid tumors with persistent moderate to severe CIPN (symptoms of numbness, tingling, or pain rated ≥4 on a numeric rating scale [NRS]) who had completed 3 or more months of chemotherapy prior to study enrollment and were not taking stable neuropathic medication were eligible. Patients were allocated 1:1:1 to real acupuncture, sham acupuncture, or usual care through computer-generated randomization conducted by the Clinical Research Database in randomly permuted blocks.

**Figure.**
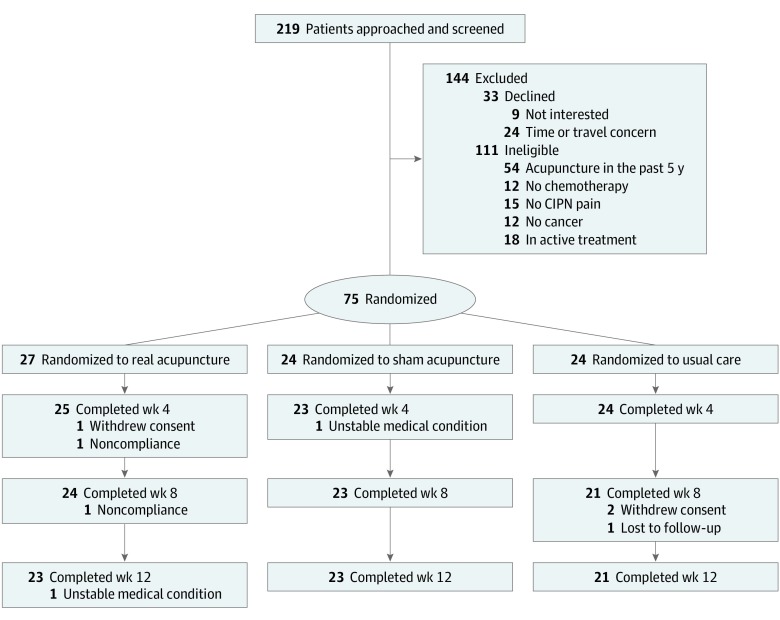
CONSORT Diagram of Participant Flow Through the Study The number of participants who completed treatment at weeks 8 and 12 were included in the analysis for those weeks. CIPN indicates chemotherapy-induced peripheral neuropathy.

The real acupuncture group received ear and body acupuncture at Shen Men, point zero, and a third electrodermal active point,^2^ and bilateral body: LI-4, PC-6, SI-3, LR-3, GB-42, ST-40, Bafeng 2, and Bafeng 3. Electrical acupuncture was also applied bilaterally from LR-3 (negative) to GB-42 (positive) at 2 to 5 Hz for 20 minutes. The sham acupuncture group received a noninsertion procedure on nonacupoints. The usual care group did not receive any interventions throughout the study period. Investigators, study coordinators, and the statistician were blinded to the treatment assignments.

The primary end point was CIPN symptom severity measured by NRS (11-point scale; 0 = no symptoms and 10 = worst symptom imaginable) at week 8. In prior studies, the NRS had high reliability and validity.^3,4^ We targeted a sample size of 25 participants in each group. This sample size allowed us to estimate the upper bound of a 1-sided 90% confidence interval for the treatment effect on outcome (eg, sham acupuncture vs real acupuncture or usual care vs real acupuncture) to 0.35-unit SDs. Mixed-effects models with an interaction term between group and assessment time (up to 8 weeks) were fit to examine whether temporal changes in a measure differed by group. The threshold for statistical significance was set at 2-sided *P* < .05.

## Results

From July 2017 to June 2018, we enrolled 75 patients with solid tumors with moderate to severe CIPN (median [interquartile range] age, 59.7 [36.3-85.9] years; 60 [80%] female; 55 [73%] white; 40 [53%] with breast cancer and 12 [16%] with colorectal cancer). In all, 24 patients were randomized to real acupuncture, 23 to sham acupuncture, and 21 to usual care. Compared with usual care, NRS-measured pain, tingling, and numbness significantly decreased in real acupuncture at week 8 (Table). From baseline to week 8, mean absolute reduction in CIPN pain was greatest in real acupuncture (−1.75 [95% CI, −2.69 to −0.81]) and least in usual care (−0.19 [95% CI, −1.13 to 0.75]) (Table). At the 8-week assessment, sham acupuncture had a reduction of −0.91 (95% CI, −2.0 to 0.18). At the longer 12-week follow-up, real acupuncture had a mean absolute reduction in NRS-measured pain of −1.74 (95% CI, −2.6 to −0.83) from baseline, while sham treatment had a reduction of −0.34 (−1.3 to 0.61). Adverse events were few and mild.

**Table.** Mean Change in Chemotherapy-Induced Peripheral Neuropathy Symptom Scores at Week 8

Symptom	Real Acupuncture (n = 24)		Sham Acupuncture (n = 23)		Usual Care, Mean (95% CI) (n = 21)
	Mean (95% CI)	*P* Value^a^	Mean (95% CI)	*P* Value^a^	
Numeric rating scale pain					
Baseline	4.15 (3.01 to 5.29)		4.12 (2.98 to 5.27)		4.75 (3.42 to 6.08)
Absolute reduction	−1.75 (−2.69 to −0.81)	.05	−0.91 (−2.00 to 0.18)	.31	−0.19 (−1.13 to 0.75)
Numeric rating scale tingling					
Baseline	5.30 (4.25 to 6.34)		5.46 (4.66 to 6.26)		5.50 (4.40 to 6.60)
Absolute reduction	−1.83 (−2.71 to −0.96)	.02	−1.22 (−2.26 to −0.17)	.18	−0.14 (−1.40 to 1.11)
Numeric rating scale numbness					
Baseline	6.59 (5.76 to 7.42)		6.25 (5.62 to 6.88)		6.25 (5.32 to 7.18)
Absolute reduction	−1.54 (−2.40 to −0.68)	.005	−1.52 (−2.56 to −0.49)	.003	0.57 (−0.62 to 1.76)

^a^ *P* values are treatment-time interaction terms computed using linear mixed-effects models, comparing with usual care.

## Discussion

We found therapeutic benefit of real acupuncture for neuropathic pain that is consistent with previous pilot acupuncture CIPN trials.^5,6^ Distinctively, our study is the first, to our knowledge, to incorporate a sham treatment and a nontreatment control to evaluate the efficacy of acupuncture for CIPN. The addition of a sham acupuncture control in an acupuncture clinical trial is difficult owing to the challenge of incorporating a truly inert placebo. In addition, a sham control limits the ability of a small effect size to elucidate a true difference between real and sham acupuncture. Not only did our study demonstrate the feasibility of conducting a sham-controlled acupuncture trial, it generated sufficient pilot data to inform a definitive sham-controlled efficacy trial. Our trial is limited by its small sample size, single center, and short-term follow-up.

In conclusion, compared with usual care, acupuncture resulted in significant improvement in CIPN symptoms. The effect size observed between real and sham control will inform a rigorous and adequately powered trial to establish the efficacy of acupuncture for CIPN.

## Notes

Supplement 1. **Trial Protocol**

Supplement 2. **Data Sharing Statement**

## References

[1] Hershman DL, Lacchetti C, Dworkin RH, ; American Society of Clinical Oncology Prevention and management of chemotherapy-induced peripheral neuropathy in survivors of adult cancers: American Society of Clinical Oncology clinical practice guideline. J Clin Oncol. 2014;32(18):-. doi:10.1200/JCO.2013.54.0914 24733808

[2] Bao T, Goloubeva O, Pelser C, A pilot study of acupuncture in treating bortezomib-induced peripheral neuropathy in patients with multiple myeloma. Integr Cancer Ther. 2014;13(5):396-404. doi:10.1177/1534735414534729 24867959 PMC4562796

[3] Downie WW, Leatham PA, Rhind VM, Wright V, Branco JA, Anderson JA Studies with pain rating scales. Ann Rheum Dis. 1978;37(4):378-381. doi:10.1136/ard.37.4.378 686873 PMC1000250

[4] Ferraz MB, Quaresma MR, Aquino LR, Atra E, Tugwell P, Goldsmith CH Reliability of pain scales in the assessment of literate and illiterate patients with rheumatoid arthritis. J Rheumatol. 1990;17(8):1022-1024. 2213777

[5] D’Alessandro EG, Nebuloni Nagy DR, de Brito CMM, Almeida EPM, Battistella LR, Cecatto RB Acupuncture for chemotherapy-induced peripheral neuropathy: a randomised controlled pilot study [published online June 29, 2019]. BMJ Support Palliat Care. doi:10.1136/bmjspcare-2018-001542 31256014

[6] Bao T, Seidman AD, Piulson L, A phase IIA trial of acupuncture to reduce chemotherapy-induced peripheral neuropathy severity during neoadjuvant or adjuvant weekly paclitaxel chemotherapy in breast cancer patients. Eur J Cancer. 2018;101:12-19. doi:10.1016/j.ejca.2018.06.008 30007894 PMC6147260

