# Supplementary material for: Effect of Acupuncture vs Sham Procedure on Chemotherapy-Induced Peripheral Neuropathy Symptoms: A Randomized Clinical Trial
Source: JAMA Netw Open. 2020 Mar 11;3(3):e200681. doi: 10.1001/jamanetworkopen.2020.0681 (PMC7066475; doi:10.1001/jamanetworkopen.2020.0681)
Supplement: Supplement 1. — Trial Protocol [file jamanetwopen-3-e200681-s001.pdf]

# Acupuncture for Persistent Chemotherapy-induced Peripheral Neuropathy Symptoms in Solid Tumor Survivors: A Pilot Study

PROTOCOL FACE PAGE FOR
MSK THERAPEUTIC/DIAGNOSTIC PROTOCOL

| | | |
|---|---|---|
| Principal Investigator/Department: | Ting Bao, MD, DABMA, MS | Medicine/Integrative Medicine |
| Co-Principal Investigator(s)/Department: | Jun Mao, MD, MSCE<br>Andrew Seidman MD | Medicine/Integrative Medicine<br>Medicine/Breast Medicine |
| Investigator(s)/Department: | Gary Deng, MD, PhD<br>Shelly Latte-Naor, MD<br>K.SimonYeung, PharmD,LAc<br>Theresa Affuso, L.Ac<br>Yi Chan, DPM, L.Ac<br>Jonathan Siman, L.Ac<br>Matthew Weitzman, L.Ac<br>Yi Lily Zhang, L.Ac<br>Andrew Vickers<br>Melissa J. Assel | Medicine/Integrative Medicine<br>Medicine/Integrative Medicine<br>Medicine/Integrative Medicine<br>Medicine/Integrative Medicine<br>Medicine/Integrative Medicine<br>Medicine/Integrative Medicine<br>Medicine/Integrative Medicine<br>Medicine/Integrative Medicine<br>Epidemiology/Biostatistics<br>Epidemiology/Biostatistics |
| Consenting Professional(s)/Department: | Ting Bao, MD, DABMA, MS<br>Jun Mao, MD, MSCE<br>Gary Deng, MD, PhD<br>Shelly Latte-Naor, MD<br>K. SimonYeung, PharmD,LAc<br>Marci Coleton, MA<br>Janice DeRito<br>Jamie Green<br>Stephanie Pearson<br>Lauren Piulson<br>Benjamin Search<br>Michelle Smith | Medicine/Integrative Medicine<br>Medicine/Integrative Medicine<br>Medicine/Integrative Medicine<br>Medicine/Integrative Medicine<br>Medicine/Integrative Medicine<br>Medicine/Integrative Medicine<br>Medicine/Integrative Medicine<br>Medicine/Integrative Medicine<br>Medicine/Integrative Medicine<br>Medicine/Integrative Medicine<br>Medicine/Integrative Medicine<br>Medicine/Integrative Medicine |

**Please Note: A Consenting Professional must have completed the mandatory Human Subjects Education and Certification Program**.

| OneMSK Sites |
|---|
| Manhattan |
| |
| |

Memorial Sloan Kettering Cancer Center
1275 York Avenue
New York, New York 10065

Table of Contents

**1.0 PROTOCOL SUMMARY AND/OR SCHEMA** ..... 3
**2.0 OBJECTIVES AND SCIENTIFIC AIMS** ..... 5
**3.0 BACKGROUND AND RATIONALE** ..... 6
**4.0 OVERVIEW OF STUDY DESIGN/INTERVENTION** ..... 8
4.1 Design ..... 8
4.2 Intervention ..... 8
**5.0 THERAPEUTIC/DIAGNOSTIC AGENTS** ..... 9
**6.0 CRITERIA FOR SUBJECT ELIGIBILITY** ..... 10
6.1 Subject Inclusion Criteria ..... 10
6.2 Subject Exclusion Criteria ..... 10
**7.0 RECRUITMENT PLAN** ..... 10
**8.0 PRETREATMENT EVALUATION** ..... 11
**9.0 TREATMENT/INTERVENTION PLAN** ..... 15
**10.0 EVALUATION DURING TREATMENT/INTERVENTION** ..... 16
**11.0 TOXICITIES/SIDE EFFECTS** ..... 16
**12.0 CRITERIA FOR THERAPEUTIC RESPONSE/OUTCOME ASSESSMENT** ..... 17
**13.0 CRITERIA FOR REMOVAL FROM STUDY** ..... 18
**14.0 BIOSTATISTICS** ..... 18
**15.0 RESEARCH PARTICIPANT REGISTRATION AND RANDOMIZATION PROCEDURES** 19
15.1 Research Participant Registration ..... 19
15.2 Randomization ..... 20
**16.0 DATA MANAGEMENT ISSUES** ..... 20
16.1 Quality Assurance ..... 21
16.2 Data and Safety Monitoring ..... 21
**17.0 PROTECTION OF HUMAN SUBJECTS** ..... 22
17.1 Privacy ..... 23
17.2 Serious Adverse Event (SAE) Reporting ..... 23
17.2.1 ..... 24
**18.0 INFORMED CONSENT PROCEDURES** ..... 25
**19.0 REFERENCES** ..... 25
**20.0 APPENDICES** ..... 29

## 1.0 PROTOCOL SUMMARY AND/OR SCHEMA

| Table 1. Protocol Summary | |
|---|---|
| Study Title | Acupuncture for Persistent Chemotherapy-induced Peripheral Neuropathy Symptoms in Solid Tumor Survivors: A Pilot Study |
| Primary Objective | To obtain preliminary data on the effects of acupuncture and sham acupuncture compared to usual care in reducing chemotherapy-induced peripheral neuropathy (CIPN) severity to help design a fully powered randomized trial. |
| Secondary Objectives | To obtain estimates of the change in CIPN severity<br>To elucidate the mechanisms of acupuncture by obtaining estimates of tactile threshold, vibration threshold, thermal threshold and controlled pain modulation.<br>To obtain estimates of the change in insomnia, anxiety, depression, and fatigue symptoms.<br>To obtain estimates of the correlation of treatment expectancy and treatment response among patients who undergo acupuncture or sham acupuncture.<br>To estimate the proportion of patients with a dose reduction and the proportion with a dose escalation of neuropathy medication during the course of the 12 weeks. |
| Patient Population | Solid tumor cancer survivors on no or stable neuropathy medication with persistent moderate to severe CIPN, defined as numbness, tingling, or pain ratings of ≥4 on the numeric rating scale (NRS), at least 3 months after neurotoxic chemotherapy completion. |
| Number of Patients Required | 75 |
| Study Design | Single-center, three – arm pilot randomized controlled trial |
| Treatment: Acupuncture Treatment Group (n=25) | Ten acupuncture treatments over the course of eight weeks, with twice weekly acupuncture treatments for the first two weeks, and then weekly treatment thereafter. |
| Treatment: Sham Acupuncture Treatment Group (n=25) | Ten sham acupuncture treatments over the course of eight weeks, with twice weekly sham acupuncture treatments for the first two weeks, and then weekly sham acupuncture treatment thereafter. |

| | |
|---|---|
| | |
| Treatment: Usual Care Group (n=25) | Twelve weeks of usual care. |
| Time to Completion | Eight weeks of treatment followed by four weeks of follow-up for a total of twelve weeks in the acupuncture and sham acupuncture arms or 12 weeks of usual care. Assessments will be conducted from baseline to four weeks after the completion of treatment. |

Figure 1: Study Schema

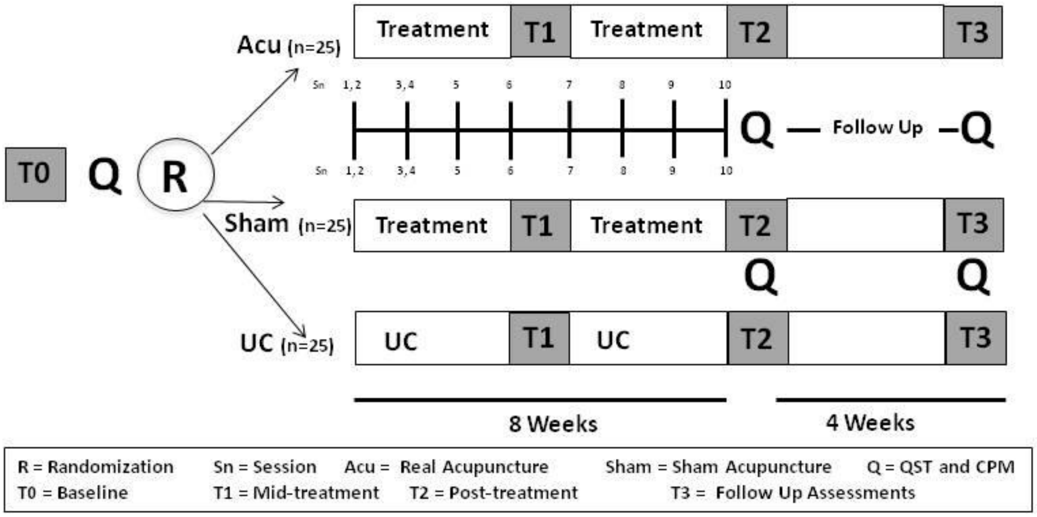


**Table 2. Study Calendar**

| Procedure | Consent/Baseline (T0)[1,5] | Week 4 (T1)[1] | Week 8 (T2)[1] | Week 12 (T3)[1] |
|---|---|---|---|---|
| Eligibility Review | X | | | |
| Height | X | | | |
| Weight | X | | | |
| Eastern Cooperative Oncology Group (ECOG) Performance Status[2,6] | X | | | |

| | | | | |
|---|---|---|---|---|
| Neuropathy Pain Scale (NPS) [6] | X | X | X | X |
| Numeric Rating Scale (NRS) [6] | X | X | X | X |
| Functional Assessment of Cancer Therapy-Gynecologic Oncology Group/Neurotoxicity (FACT-GOG/NTX ) questionnaire[6] | X | X | X | X |
| CIPN severity assessed by NCI-CTC 4.0[6] | X | | X | X |
| Insomnia Severity Index (ISI) [6] | X | | X | X |
| Hospital Anxiety and Depression Scale (HADS) [6] | X | | X | X |
| Brief Fatigue Inventory (BFI) [6] | X | | X | X |
| Treatment Expectancy Scale[6] | X | X | X | X |
| Study Allocation Belief [6] | | | | X |
| Pain Medication List[3, 6] | X | X | X | X |
| Safety Assessment[4] | X | X | X | |
| Quantitative Sensory Testing (QST)[5] | X | | X | X |
| Controlled Pain Modulation (CPM)[5] | X | | X | X |

1. In the case of any missed sessions during the study, the testing, questionnaires and other assessments should occur in the same week as planned. In the case of early discontinuation of treatment, all attempts will be made to collect the questionnaires until the end of the study.
2. ECOG Performance Status will be assessed ≤ 28 days before the consent.
3. The medication lists will include both prescription and non-prescription pain medication.
4. Patients will be monitored for side effects at each acupuncture and sham acupuncture session .
5. This will be performed by an IMS research study assistant who will go through training before the initiation of the study. A window of + 1 week applies to the baseline assessments, and a window of +/- 3 days applies to week 8 and week 12 assessments.
6. Can be performed over the phone.

## 2.0 OBJECTIVES AND SCIENTIFIC AIMS

**Primary Objective:**

To obtain preliminary data on the effects of acupuncture and sham acupuncture compared to usual care in reducing CIPN severity to help design a fully powered randomized trial.

**Secondary Objectives:**

- To obtain estimates of the change in chemotherapy-induced peripheral neuropathy (CIPN) severity
- To elucidate the mechanisms of acupuncture by obtaining estimates of tactile threshold, vibration threshold, thermal threshold and controlled pain modulation. To obtain estimates of the change in insomnia, anxiety and depression, and fatigue symptoms.
- To obtain estimates of the correlation of treatment expectancy and treatment response among patients who undergo acupuncture or sham acupuncture.
- To estimate the proportion of patients with a dose reduction and the proportion with a dose escalation of neuropathy medication during the course of the 12 weeks.

## 3.0 BACKGROUND AND RATIONALE

In 2016 alone, an estimated 1.5 million people in the U.S. will be diagnosed with solid tumor,[1] and may require chemotherapy. Many chemotherapy agents such as platinums and taxanes that are commonly used to treat solid tumor may cause persistent chemotherapy-induced peripheral neuropathy (CIPN)[2,3]. With advanced medical treatment, the majority of solid tumor patients may survive and become long-term cancer survivors. Up to 55% of such patients may suffer from persistent CIPN long after chemotherapy completion.[4,5] Although the mechanisms of CIPN have not been fully elucidated, proposed mechanisms include damage to axon, dorsal root ganglia or sensory, motor, and autonomic nerve fibers.[6] CIPN primarily presents as distal, symmetrical sensory neuropathy, with motor symptoms rarely present until severe sensory deficit occurs. CIPN symptoms usually include paresthesia (tingling, burning sensation), hyperalgesia (increased sensitivity to noxious stimulation), and allodynia (pain from normally innocuous stimulation), resulting in significantly worsened quality of life.[7,8]

Treatment for CIPN has been limited to symptomatic management with narcotics, antidepressants, anticonvulsants, and vitamins, although data supporting their efficacy are limited.[9] Studies suggest that analgesic regimens typically produce only modest pain relief and have common side effects such as dizziness, sedation, dry mouth, and constipation.[7] Antidepressants such as nortriptyline and antiepileptics such as gabapentin and lamotrigine have been reported to be effective in reducing neuropathic symptoms associated with various other etiologies. However, none has been shown to be effective in reducing CIPN symptoms.[10-12] The only successful clinical trial for the treatment of painful CIPN showed that at 30 mg daily for 1 week followed by 60 mg daily for 4 more weeks, the antidepressant duloxetine caused a modest 1.06 point reduction in pain on a 10-point scale from baseline compared with a 0.34 point reduction for placebo.[13] However, duloxetine use was also associated with side effects including fatigue and nausea, as well as a 12% dropout rate vs. 1% for placebo.[13] Subgroup analysis also suggested that the effectiveness of duloxetine might have been limited to those with oxaliplatin-induced peripheral neuropathy,[13] leaving an additional gap in

treatment options for other cancer survivors suffering from CIPN. As such, the 2014 American Society of Clinical Oncology CIPN guidelines stated a “moderate recommendation for treatment with duloxetine” and recommended further research in this area.[14] The FDA has not approved duloxetine for the treatment of CIPN.[15] Consequently, new safe and effective treatments are needed.

Preliminary studies indicate that acupuncture is safe and reduces diabetes- and HIV-associated PN symptoms.[16-18] Two single-arm pilot studies have examined acupuncture’s ability to reduce symptoms of CIPN in multiple myeloma (MM) patients. Our group conducted a pilot study of 27 MM patients with moderate to severe bortezomib-induced PN that demonstrated significantly reduced CIPN pain (by an average 28 points on a 100-point scale), and improved function following 10 weeks of acupuncture.[19] Another study of 19 MM patients showed that acupuncture was safe and possibly effective in treating thalidomide/bortezomib-induced PN by significantly improving FACT/GOG-Ntx scores after an intensive 9-week acupuncture treatment.[20] The pre-post change in CIPN symptoms in our prior study was more substantial than that reported for duloxetine. There were no significant side effects associated with acupuncture in either study. As such, acupuncture has emerged as a promising non-pharmacologic approach to treat CIPN. Additional randomized controlled trials comparing acupuncture with placebo and usual care are needed to further evaluate the efficacy of acupuncture in treating CIPN. The proposed pilot phase II trial will help to establish the effect size of acupuncture and power calculation to design a follow-up definitive clinical trial to further evaluate the role of acupuncture in reducing CIPN symptoms.

The Nerve Conduction Study (NCS) has been used to diagnose CIPN and monitor treatment response. However, because the NCS is ineffective in detecting small fiber neuropathy, it may not be effective in making the diagnosis of CIPN or in monitoring treatment response.[21] Indeed, NCS may be normal in certain types of peripheral neuropathy as small sensory fibers are involved [6] and do not correlate with subjective findings in oxaliplatin-induced peripheral neuropathy[22]. Therefore, an objective measurement of small fiber neuropathy is needed. Quantitative Sensory Testing **(**QST) is an emerging clinical and translational tool to measure small sensory fiber impairment. It quantifies somatosensory function based on measurements of responses to calibrated, graded innocuous or noxious stimuli.[23] QST studies have shown that both tactile and vibration sensation abnormalities have been detected in patients with diabetic peripheral neuropathy, similar to the involvement of small nerve fibers in CIPN.[24] A prior study showed that changes in CIPN symptoms, such as hand and foot numbness/discomfort, were significantly associated with changes in the vibration threshold.[25] These studies provide the initial evidence that QST may help better phenotype CIPN and serve as an objective marker of somatosensory function recovery. We propose to incorporate a QST protocol in our trial to: 1) describe the sensory phenotypes of patients with CIPN; and 2) evaluate the effect of acupuncture on QST measures. By doing so, we may establish QST as a reliable objective measurement of CIPN severity and treatment response, further elicit the underlying pathophysiology of CIPN, and identify effective treatment strategies.

Although the exact mechanism of acupuncture is not fully understood, it has been proposed that acupuncture may work through augmenting descending inhibitory pain control.[26] Animal studies suggest that acupuncture works by stimulating Aβ, Aδ, and C afferent fibers via needle insertion, which then activate the descending inhibition from supra-spinal and higher centers,[27] and subsequently lead to endogenous pain inhibition[28]. Correlative clinical studies in humans to confirm

this theory are lacking. The Conditioned Pain Modulation (CPM) protocol has been used to assess the intactness of the descending pain inhibition pathway.[29,30] Decreased pain inhibition has been found in many patients with idiopathic pain syndrome.[31-35] CPM has been shown to predict the efficacy of duloxetine in treating painful diabetic neuropathy as patients with ineffective CPM benefit more from duloxetine.[36] We propose incorporating a CPM protocol into our trial to: 1) describe the CPM pattern in patients with CIPN; 2) explore whether baseline CPM patterns predict response to acupuncture in CIPN patients; and 3) explore if acupuncture treatment improves CPM. This will help us better understand a mechanism of acupuncture analgesia and advance the field of acupuncture and CIPN research.

**Preliminary Data**

Our group has completed a single-center, single-arm pilot study to evaluate the safety, feasibility, and efficacy of 10 weeks of acupuncture to treat bortezomib-induced PN in multiple myeloma patients.[19] Twenty-seven multiple myeloma patients experienced moderate to severe CIPN despite adequate medical intervention and chemotherapy discontinuation (median 19 months). At the end of the 10 weeks, compared to baseline, patients experienced significantly reduced CIPN pain with 28 points of pain reduction on a 100-point scale ($p < 0.0001$ at weeks 10 and 14). Patients also experienced improved function and better QoL without any significant side effects.[19] Our group has tested the proposed sham acupuncture protocol on four patients with moderate CIPN and learned that these patients were well blinded to the treatment assignment. This demonstrates the feasibility of conducting a sham acupuncture-controlled trial in this setting.

## 4.0 OVERVIEW OF STUDY DESIGN/INTERVENTION

### 4.1 Design

This is a single-center, three-arm, phase IIB, pilot randomized controlled trial comparing acupuncture, sham acupuncture and usual care. We will enroll 75 solid tumor cancer survivors who completed chemotherapy at least 3 months prior who have persistent moderate to severe CIPN symptoms defined as at least one NRS≥4 (25 patients in each arm). With a total sample size of 75 this trial will provide preliminary data on the effects of treating patients with acupuncture to reduce persistent CIPN as measured by NRS and other CIPN measurements such as NPS and FACT/GOG-Ntx when compared to sham acupuncture or usual care. The primary end point of this study is NRS of the patients' most bothersome CIPN symptom (tingling, or numbness or pain) at week 8. It is the CIPN symptom having the maximum NRS measured at baseline. If more than one symptom share the maximum value, we would ask the patient to pinpoint the most bothersome symptom and use that one as the primary endpoint. Secondary end points are NPS, FACT/GOG-Ntx, ISI, HADS, BFI, TES, QST and CPM. These data will then be used in the design of a fully powered trial.

### 4.2 Intervention

Subjects will be stratified based on the type of the most bothersome CIPN symptom (tingling vs numbness vs pain) and severity of such symptom (moderate 4-6 vs 7-10 on NRS) first. They will then be randomized at a 1:1:1 ratio to the acupuncture group (ACU), sham acupuncture group (SHAM) or usual care group (UC). Integrative Medicine Service acupuncturists will perform the acupuncture

and sham acupuncture treatments. The acupuncture group and sham acupuncture group will undergo 8 weeks of outpatient acupuncture or sham acupuncture treatments (twice weekly for 2 weeks, then weekly for 6 weeks, 10 treatments in total). We have designed this approach to standardize acupuncture treatment for future broad use; it is based on the understanding that CIPN is a global symptom complex rather than individual isolated or local symptoms. We will encourage all patients to keep their pain medication regimen unchanged during the 8-week intervention and will record all pain medication usage during the study. A window of ± 3 days applies to acupuncture sessions. A missed session will not be made up without prior approval from the PI. Highly experienced and licensed acupuncturists who are part of MSK's Integrative Medicine Service will perform the acupuncture or sham acupuncture treatments.

**<u>Acupuncture Group:</u>** Acupuncturists will treat patients at bilateral Shen Men ear points, at one additional auricular acupoint where an electrodermal signal is detected,[19] and at bilateral body acupoints LI-4, LI-11, PC-6, SI-3, LR-3, GB-43, ST-40, Bafeng 2, and Bafeng 3. Acupuncturists will have the option of not inserting needles in the extremities that have no symptoms. These acupoints were selected based on our clinical experience and prior research.[37,38] After disinfecting the skin with an alcohol swab, the acupuncturist will insert sterilized 0.16 mm x 15 mm disposable filiform acupuncture needles in the ear points; 0.25 mm x 30 or 40 mm needles of the same quality will be used on body points. The acupuncturist will insert needles approximately 12.5 mm (0.5 inch) into the skin for body points and 2.0 mm for ear points. Needles will remain for 30 minutes after the patient indicates experiencing *de qi* sensation (feeling of soreness, numbness, or distention) at LI-4, SI-3, and ST-40 points. In addition, electrical stimulation will be applied bilaterally as follows: from LI4 (negative) to LI11 (positive), and from LR-3 (negative) to GB-43 (positive) at 2–5 Hz for 30 minutes. The current varies based on the patient's response and aims at providing constant gentle stimulation, and ranges from 0-40mA. This is very similar to the EA protocol used in the 2014 pilot study by Garcia et al.[20] All points used will be documented.

**<u>Sham Acupuncture Group:</u>** For the sham treatment, we will use a combination of non-acupuncture points and a non-insertion procedure from a previously validated placebo acupuncture method.[39,40] Acupuncturists will tap an empty plastic needle guiding tube on the bony area close to but away from each of the 8 real acupoints in the arm and leg (LI4, SJ5, LI11, ST40) to produce some discernible sensation and then immediately apply a needle with a piece of adhesive tape to the dermal surface, without needle insertion, for a total of 30 minutes. The EA device will be attached to the needles without being turned on. Subjects in this group will receive the sham acupuncture procedure on the same schedule as subjects in the real acupuncture group. In both groups, patients' eyes will be covered with patches so they cannot observe the treatment procedure. To improve incentive to join and stay in the study, we will unblind all patients at Week 12, and offer those randomized to the Sham group 8 sessions over 8 weeks of Acu (real) treatment. All points used will be documented.

**<u>Usual Care Group:</u>** We will ask patients randomized to UC to come to our clinic three times during the study, at Baseline, Week 8, and Week 12 to undergo QST and CPM tests. We will ask them to complete all questionnaires (CIPN severity by 0-10 11-point numeric rating scale, FACT/GOG-Ntx-11, NPS) and their pain medication use at these time points. Week 4 questionnaires can be completed over the phone. As an incentive for patients to join the study - and for those randomized to control to stay in the study - controls will be offered 8 sessions over 8 weeks of Acu (real) treatment once they have completed Week 12 assessments.

## 5.0 THERAPEUTIC/DIAGNOSTIC AGENTS

Acupuncture treatment will be given by certified, licensed MSK staff acupuncturists. All have extensive clinical experience in treating cancer patients.

**Acupuncture Needles**: Seirin acupuncture needles will be used in this study. The needles are purchased and distributed from Seirin® in the United States (http://www.seirinamerica.com). Seirin acupuncture needles are approved by the FDA (http://www.accessdata.fda.gov/cdrh_docs/pdf/K962809.pdf).

## 6.0 CRITERIA FOR SUBJECT ELIGIBILITY

### 6.1 Subject Inclusion Criteria

- English-speaking
- Age ≥ 18 years old
- Solid tumor survivors with no evidence of disease
- Moderate to severe CIPN, defined by symptoms such as numbness, tingling, or pain ratings of 4 or greater on a 0-10 numeric rating scale (NRS)
- Have completed neurotoxic chemotherapy at least 3 months prior to enrollment
- If taking anti-neuropathy medications, they are on a stable regimen (no change in 3 months)

### 6.2 Subject Exclusion Criteria

- Patients with a pacemaker
- Prior acupuncture treatment within 5 years of enrollment

## 7.0 RECRUITMENT PLAN

Our primary recruitment approach will be via sending recruitment letters to potential participants. Potential patients who meet basic eligibility criteria will be identified via querying Dataline and sent a recruitment letter. The recruitment letter will introduce the study to potential participants and state that we are conducting a study of acupuncture for neuropathy and if interested in learning more about the study, the patient should contact the research study assistant. The letter provides patients with an opt-out option if they do not wish to participate or be contacted further. We have successfully used this recruitment method for similar studies.

In addition to sending recruitment letters, potential participants also can be identified and referred to the study RSA by protocol investigators or other MSK healthcare providers or can be self-referred. The study PI and other members of the research team will reach out to colleagues about the study and present at Service meetings. Information about the protocol will also be provided in lay language on MSKCC's web site and on clinicaltrials.gov. Additionally, printed materials will be posted in clinic areas where we have successfully posted study materials for other Integrative Medicine studies before (e.g., MSK's solid tumor clinics, cancer survivorship clinic, and integrative

medicine clinic). Permission from the clinic sites will be obtained before posting in any location. All study recruitment materials will be submitted to, and approved by, the Institutional Review Board. We have used these recruitment strategies successfully to recruit participants to similar studies.

Integrative Medicine Research Study Assistants (RSAs) will screen potential interested and eligible patients, and schedule screening and consenting appointments. A study investigator will confirm eligibility. A study investigator or RSA specially trained for participation in the study will obtain informed consent. After establishing eligibility, the study staff will register participants.

Initial contact with prospective subjects typically will be made by a member of the study team. The recruitment process presents no more than minimal risk to patient privacy, and minimal PHI will be maintained on screening logs. For these reasons, we seek a (partial) limited waiver of authorization to: (1) review medical records to identify potential research subjects and obtain information relevant to the enrollment process; (2) converse with patients regarding possible enrollment; (3) handle PHI contained in those records and provided by potential subjects; and (4) maintain minimal PHI information in a screening log of patients approached.

## 8.0 PRETREATMENT EVALUATION

Attendings will approve potential patients per eligibility requirements noted above. Baseline measures to assess CIPN severity will be conducted by a chemotherapy nurse, research nurse, or physician. At baseline, the following information will be collected.

- ECOG Performance Status
- Severity of CIPN as defined by NCI-CTC 4.0
- FACT-GOG/NTx questionnaire
- Neuropathy Pain Scale (NPS)
- Numeric Rating Scale (NRS)
- Treatment Expectancy Scale (TES)
- Pain medication list
- Quantitative Sensory Testing (QST)
- Controlled Pain Modulation (CPM)
- Insomnia Severity Index (ISI)
- Hospital Anxiety and Depression Scale (HADS)
- Brief Fatigue Inventory (BFI)

**Numeric Rating Scale (NRS)**

The primary endpoint will be measured by NRS of the most bothersome CIPN symptom. The numeric rating scale is an 11-point numeric scale with 0 representing no symptoms and 10 representing the worst symptoms imaginable. The NRS has been widely used to measure severity of symptoms, especially pain[41,42]. Its high reliability and validity has been observed in prior studies[43,44]. In clinical trials for multiple conditions, i.e. diabetic neuropathy, chronic low back pain, a reduction of 2 points or 30% on the NRS scores has been shown to be clinically significant[42]. The NRS is a valid and reliable scale to measure CIPN symptoms (pain, tingling, numbness) intensity. It

is simple and can be administered both verbally and in writing, and is effective as an initial screening tool to assess patient's eligibility for the trial. The components of this measurement can be found in the Appendix A. It will be collected at baseline and at weeks 4, 8, and 12 for the acupuncture group and sham acupuncture group. The usual care control group will complete the questionnaire at baseline and weeks 4, 8 and 12. It takes no more than 2 minutes to complete. Week 4 questionnaires for the usual care group may be completed over the phone.

**NCI –CTC 4.0**

The National Cancer Institute (NCI) of the National Institutes of Health (NIH) has published standardized definitions for adverse events (AEs), known as the Common Terminology Criteria for Adverse Events (CTCAE, also called "common toxicity criteria"), to describe the severity of organ toxicity for patients receiving cancer therapy. Toxicity is graded as mild (Grade 1), moderate (Grade 2), severe (Grade 3), or life-threatening (Grade 4), with specific parameters according to the organ system involved. Death (Grade 5) is used for some of the criteria to denote a fatality. Severity of CIPN defined by NCI-CTC 4.0 is detailed in Appendix B. CIPN severity will be assessed at baseline and weeks 8 and 12. The CIPN grade for the baseline measure can be obtained from the patients EMR if it was measured within two weeks of the consent/baseline visit.

**FACT/GOG-Ntx**

The FACT/GOG-Ntx questionnaire is comprised of the FACT-G plus the 11-item Ntx subscale. Only the 11 items from the Ntx subscale of the FACT/GOG-Ntx questionnaire will be administered in this study. The 11-item neurotoxicity subscale covering sensory neuropathy, motor neuropathy, hearing neuropathy, and dysfunction associated with neuropathy to assess the details of neurotoxicity symptoms in the patient. It correlates with meaningful clinical changes in neuropathy and takes about 2 minutes to finish. The FACT/GOG-Ntx has demonstrated reliability and validity, with a Cronbach's alpha of 0.81 for the neurotoxicity subscale and an overall Cronbach's alpha of 0.84.[45] Further, the FACT/GOG-Ntx has demonstrated sensitivity to meaningful clinical distinctions and change over time.[45] Higher scores on the Ntx subscale of the FACT/GOG-Ntx represents higher functioning and fewer/less severe CIPN symptoms. The FACT/GOG-Ntx has been validated for self, interviewer, and computer administration. Appendix A lists the components of this measurement. It will be collected at baseline and at weeks 4, 8, and 12 for the acupuncture group and sham acupuncture group. The usual care control group also will complete the questionnaire at baseline, week 4, weeks 8 and 12. Week 4 questionnaires for the usual care group may be completed over the phone.

**Neuropathic Pain Scale (NPS)**

The Neuropathic Pain Scale (NPS) is a multidimensional tool that uses self-report visual analogue scales (VAS) to quantify, on a scale of 0-10, global pain intensity and unpleasantness and eight other descriptive qualities of neuropathic pain. The NPS also includes one semi-structured question about temporal sequence.[46,47] It takes no more than 2 minutes to complete. Each of the individual items in the NPS serves as a stand alone score. All 10 of the items are relevant to the goals of the study and will be summed up to produce a total score. The NPS is capable of distinguishing neuropathic pain from non-neuropathic pain.[48] The 10 items demonstrate weak correlations with one another, supporting their discriminate validity.[46] Appendix A lists the components of this

measurement. It will be collected at baseline and at weeks 4, 8, and 12 for the acupuncture group and sham acupuncture group. The usual care control group will complete the questionnaire at baseline and weeks 4, 8 and 12. It takes no more than 2 minutes to complete. Week 4 questionnaires for the usual care group may be completed over the phone.

**Treatment Expectancy Scale (TES)**

Pre-randomization treatment expectancy will be assessed using a validated measure. The Treatment Expectancy Scale (TES) is a 4-item instrument developed by Mao (co-PI) et al. TES is scored by adding up the score of each 4 items, with 1 indicating "Not at all agree" and 5 indicating "Completely agree" with the expected improvement as result of acupuncture. The score of instrument ranged from 4 to 20 out of a possible 4 to 20, with higher scores indicating greater expectancy. Outcome expectancy has long been considered an important predictor of treatment outcomes[49] and has gained important consideration in acupuncture in recent years.[50] It has demonstrated reliability (Cronbach's α of 0.82) and validity and is positively correlated with patient self-reported efficacy and satisfaction.[51] The TES has also been validated in breast cancer survivors and is sensitive to change over time in response to acupuncture treatment.[52] In our previous study, we found expectancy was stable overtime in the waitlist control group.[53] Baseline expectancy predicted treatment outcomes of acupuncture. The TES will also be administered at weeks 4, 8, and 12. (Appendix A)

**Pain Medication List**

The pain medication list will be collected at baseline, and weeks 4, 8, and 12. The information can be captured via a phone call for the usual care group at week 4. The form lists the name and dosage of the neuropathy medication the patient is taking, if any. The lists will be compared before and at week 8 to determine if the medication was dose reduced or escalated. If the dosage or frequency resulted in total daily dosage increases, then it will be considered dose escalation. If the total daily dosage decreases, then it will be considered dose reduction. (Appendix D)

**Quantitative Sensory Testing (QST)**

At weeks 0 (Baseline), 8 and 12, all participants will receive QST from a trained research study assistant (RSA). To minimize inter-observer variability in measurement,[54] the same RSA will conduct all pre- and post-measurements for participants when possible. This RSA will be blinded to treatment allocation. Participants will be asked not to take any pain, stimulant, or sedative medication for at least 12 hours prior to the testing session. We will conduct QST in a quiet room with little environmental stimuli to detect tactile threshold (TT), vibration threshold (VT), and thermal threshold (THT). The TT and pressure pain threshold will be evaluated by means of Von Frey's filaments (Somedic; Stockholm, Sweden), a set of 17 filaments with hairs of different diameters. We will ask the subject to sit in a comfortable position and close their eyes so that the examination is not influenced by visual input. TT will be assessed at the dorsum of the distal interphalangeal joint of the right- and left- middle fingers and both the left and right big toes, as described previously.[55] The VTs will be assessed using a handheld biothesiometer (Bio-Medical Instrument Company; Newbury, Ohio), whose amplitude may be gradually increased until the threshold of vibratory sensation is reached.[56] The VTs will be evaluated at the dorsum of the distal interphalangeal joints of the right

and left index fingers and both the left and right big toes. We will ask subjects to indicate when they feel the vibration stimulus for the first time (perception threshold) and when this stimulus disappears again (disappearance threshold). The average of three-paired measurements is considered to be the VT at the location investigated. The thermal threshold will be measured by the TSA-II system, which is a thermal sensory analyzer, providing for evaluation of cool sensation, warm sensation, heat-pain, and cold-pain thresholds. Its temperature (stimulation) range is 0 to 50$^{o}$C, with a programmable stimulation rate of up to 8$^{o}$C per second. Thermal thresholds will be assessed by the method of limits.[57] First, the thermode will be attached and the skin will be allowed to adapt to a temperature of 32°C. For the sensory threshold assessment, the thermode will be warmed or cooled at a slow rate (1°C/s) until the subject perceives the first warm or cold pain sensation. For the heat-pain and cold- pain thresholds, we will increase/decrease the temperature at a rate of 2°C/s until the moment that the subject indicates the transition of the warm/cold sensation into a painful sensation. We will repeat each test three times and average the results to obtain the threshold value.

**Controlled Pain Modulation (CPM)**

At baseline, at the end of the 8-week acupuncture treatment, and at week 12, all patients will undergo CPM testing to determine the effectiveness of their descending inhibitory pathway. We will use the above-mentioned TSA-II system to administer tonic heat pain as the “test stimulus” in the CPM protocol. Hand immersion in the water bath apparatus, a temperature controlled container, will serve as a conditioning stimulus. We will use Heto water bath for hand immersion as the conditioning stimulus[36]. We will measure CPM using the parallel paradigm in which two identical noxious ‘test stimuli’ will be delivered before and simultaneously with a noxious ‘conditioning stimulus.’ The test stimulus is first applied to the dominant forearm to reach a pain rating of greater than 60 on a numeric pain scale of 0-100. Patients will be asked to rate the level of pain intensity of the test stimulus (pain60 temperature) every 10 seconds (overall 4 pain reports). After a 15-minute break, the non-dominant hand will be immersed in the cold water bath up to the wrist at 10$^{o}$C for 60 seconds. During the last 30 seconds of this immersion, the test stimulus will be repeated, and the patient will again rate the level of pain intensity experienced. The CPM effect is calculated as the difference (last minus first) in the average pain scores of the 2 test stimuli. A negative value indicates efficient CPM. This paradigm of conditioned pain modulation is routinely used in pain research[36,58].

**Insomnia Severity Index (ISI)**

We will use patient-reported insomnia severity as measured by the Insomnia Severity Index (ISI) as a secondary objective measure. The ISI is one of the few well-validated patient-reported outcome measures designed to specifically assess the impact on daytime functioning and the amount of associated distress.[59] The ISI includes 7 items that are scored on a five-point scale ranging from 0 to 4 with higher scores representing more severe insomnia symptoms. The optimal cutoff scores are 0-7 (no clinically significant sleep difficulties, 8-14 (sleep difficulties warrant further investigation) and 15+ (presence of clinically significant insomnia).[59] The ISI has demonstrated internal consistency, reliability, construct validity, specificity and sensitivity in a representative sample of 1670 cancer

patients.[60] The ISI has established minimally important change values to ensure that the change is not only statistically, but also clinically, meaningful to patients.[60] A reduction of eight points has been deemed to be clinically significant improvement.[60] The ISI has been used in several insomnia trials in cancer survivors and has demonstrated sensitivity to change in response to intervention as well as discriminating effects between interventions. All groups will complete the questionnaire at baseline and weeks 8 and 12. (Appendix A)

**Hospital Anxiety and Depression Scale (HADS)**

A 14-item, self-rated instrument for anxiety (7 items) and depression (7 items) symptoms in the past week and has been extensively used in people with cancer.[61] Established cutoffs for the depression and anxiety scales are independent and are: 0–7 not significant; 8–10 subclinical; and 11-21 clinically significant depression/anxiety. All groups will complete the questionnaire at baseline and weeks 8 and 12. (Appendix A)

**Brief Fatigue Inventory (BFI)**

The BFI has been shown to have good reliability and to correlate well with other measures of fatigue.[62] The three items rate current level of fatigue, and worst and usual fatigue in the prior 24 hours. The BFI score is calculated from the mean of completed items. All groups will complete the questionnaire at baseline and weeks 8 and 12. (Appendix A)

## 9.0 TREATMENT/INTERVENTION PLAN

Patients will be randomized to one of three treatment groups, detailed below. Acupuncture treatments will be given at the Integrative Medicine Outpatient Center on 74th Street and First Avenue and/or at MSK's Breast and Imaging Center (300 East 66th Street, between First and Second Avenues) and/or at MSK's Brooklyn Infusion Center (557 Atlantic Avenue, between 3rd and 4th Avenues). Patients who choose to receive acupuncture treatments at a non-Manhattan location will need to come into Manhattan for baseline, week 8, and week 12 assessments. Each treatment session will be approximately 30 minutes in duration, 10 minutes to insert needles, 20 minutes to leave needles in. Patients will receive twice weekly treatment each week for the first two weeks, followed by six weekly treatments, for a total of ten treatments over eight weeks. Adverse effects related to the administration of acupuncture will be collected each week before and after each treatment by the acupuncturist or research study assistant . All Integrative Medicine Service acupuncturists are licensed, credentialed employees of MSK. In both groups, patients' eyes will be covered with patches so they cannot observe the treatment procedure. To improve incentive to join and stay in the study, we will unblind all patients at Week 12, and offer those randomized to the Sham and UC groups 8 sessions over 8 weeks of Acu (real) treatment. For acupuncture points, please refer to section 4.2.

**Acupuncture Group:** After disinfecting the skin with an alcohol swab, the acupuncturist will insert sterilized 0.16 mm x 15 mm disposable filiform acupuncture needles in the ear points; 0.25 mm x 40 mm needles of the same quality will be used on body points. The acupuncturist will insert needles 12.5 mm (0.5 inch) into the skin for body points and 2.0 mm for ear points. Needles will remain for 30 minutes after the patient indicates experiencing *de qi* sensation.

**<u>Sham Acupuncture Group:</u>** For the sham treatment, we will use a combination of non-acupuncture points and a non-insertion procedure from a previously validated placebo acupuncture method.[39,40] Acupuncturists will tap an empty plastic needle guiding tube on the bony area next to each of the 8 real acupoints in the arm and leg (LI4, SJ5, LI11, ST40) to produce some discernible sensation and then immediately apply a needle with a piece of adhesive tape to the dermal surface, without needle insertion,for a total of 30 minutes

**<u>UC group:</u>** We will ask patients randomized to UC to come to our clinic three times during the study, at Baseline, Week 8, and Week 12 to undergo QST and CPM tests. We will ask them to complete all questionnaires (CIPN severity by 0-10 11-point numeric rating scale, FACT/GOG-Ntx-11, NPS) and their pain medication diary at these time points.  Questionnaires at Week 4 can be completed over the phone.

## 10.0 EVALUATION DURING TREATMENT/INTERVENTION

The study schema and study calendar are presented in Section 1.0., Figure 1 and Table 2, respectively. Validated patient-reported outcome questionnaires: Functional Assessment of Cancer Therapy/ Gynecologic Oncology Group-Neurotoxicity (FACT/GOG-Ntx) questionnaire, Neuropathy Pain Scale (NPS), and Numeric Rating Scale (NRS) for pain, tingling and numbness will be assessed at baseline, and weeks 4, 8, and 12 for the acupuncture and sham acupuncture groups. The Treatment Expectancy Scale (TES) will be completed at baseline, weeks 4, 8, and 12. The usual care control group will complete the questionnaires at baseline, and weeks 4, 8 and 12. CIPN severity will be assessed at baseline and weeks 8 and 12. The pain medication list will be collected at baseline, and weeks 4, 8, and 12.  Both the quantitative sensory testing and Controlled Pain Modulation (CPM) will occur at baseline (+ 1 week), at the end of the 8-week treatment phase (+/-3 days), and at 12 weeks (+/-3days). All groups will complete the Insomnia Severity Index (ISI), Hospital Anxiety and Depression Scale (HADS), and Brief Fatigue Inventory (BFI) at baseline, and weeks 8 and 12.  At the end of the study, patients in the true and sham acupuncture groups will complete the Study Allocation Belief Form,  to guess which group they were assigned for evaluation of blinding to random assignment and why they believe that to be the case.

## 11.0 TOXICITIES/SIDE EFFECTS

Patients will be monitored for side effects at each visit.  Only adverse effects related to the administration of acupuncture will be collected each week by the acupuncturist or research study assistant.

Acupuncture is a generally safe treatment modality when performed by qualified practitioners.  No serious adverse events were reported in our pilot study.

<u>Acupuncture:</u>

Most common side effects (<5%):

- Very minor bleeding at acupuncture sites
- Bruises at acupuncture sites

- Minor pain or an unfamiliar sensation at acupuncture sites (local or radiating)

Less common side effects (<1%):

- Local allergic reaction (urticaria)
- Drowsiness, sleep disturbances (insomnia)
- Anxiety, nervousness
- Fainting, dizziness, nausea, vomiting (vasovagal reaction)

Rare but more serious side effects (<0.1%):

- Local skin infection

Severity will be graded as “serious” or non-serious.” Serious AEs are those that require hospitalization, lead to death or disability or require urgent medical attention to prevent death or disability. The intensity of non-serious AEs will be graded as mild, moderate or severe.

Causality will be assessed as follows. The AE will be described as "Probable" if all of the following apply: there is a rational relationship between the occurrence of the AE and the time of treatment; the AE has already been described as an AE of acupuncture or could reasonably be anticipated to be an acupuncture AE; regression or disappearance of the AE (unless permanent) after discontinuation of treatment or dose reduction; AE cannot plausibly be explained in terms of other causal factors. The AE will be described as "Possible" if all of the following apply: there is a rational relationship to the time of treatment administration; the AE has already been described as an AE of acupuncture; the AE could be explained by other factors. The AE will be described as "Improbable" if the following apply: there is no rational relationship to the time of treatment administration; the AE has not been reported so far as a side effect of treatment and cannot reasonably be expected as an acupuncture side-effect and any of the following: non-permanent AE persists after discontinuation of the treatment or dose reduction; repeated exposure does not lead to reappearance of the AE; AE could be explained by numerous other factors. The AE will be described as "No relationship" if all of the following apply: there is no rational relationship to the time of treatment administration; AE is evidently caused by other factors, e.g. symptom of a concomitant disease. The AE will be described as "Unable to evaluate" if the amount and content of data do not permit a judgment of the relationship to the treatment.

Quantitative Sensory Testing (QST): QST is not invasive and associated with no known risk.

Controlled Pain Modulation: CPM is not invasive and associated with no known risk.

Risk of psychological distress: Some of the questions in the questionnaire may elicit distress for some patients. If a patient demonstrates clinically significant distress, s/he will be referred to the appropriate clinical/psychosocial services. An appropriate referral will be made if anxiety (7 items) or depression (7 items) scores for the HADS is greater than 10 as 11-21 indicates clinically significant depression/anxiety.

## 12.0 CRITERIA FOR THERAPEUTIC RESPONSE/OUTCOME ASSESSMENT

The primary endpoint will be measured by NRS of the most bothersome CIPN symptom and measured at time of consent (baseline) and at 8 weeks, after the final acupuncture session for the acupuncture or sham acupuncture arms and at baseline and after 8 weeks of usual care for patients randomized to the usual care arm. Secondary endpoints will be measured by the FACT/GOG-NTx, NPS, NCI-CTC 4.0, ISI, HADS, BFI, TES, QST, and CPM. Additionally, NRS will be assessed as a secondary aim at baseline, 4 weeks, and 12 weeks for all arms. All non-NRS secondary endpoints will be assessed at baseline, 8 weeks and 12 weeks. The NPS and FACT-GOG/NTX will also be assessed at 4 weeks.

All patients enrolled in the study who completed acupuncture or sham acupuncture will be included in the analyses, and any patients who withdraws from the study or who is lost to followup will be described.

## 13.0 CRITERIA FOR REMOVAL FROM STUDY

Patients will be removed from the study if they experience unacceptable adverse events from acupuncture. If at any time the primary oncologist believes it is in the best interest of the participant to be withdrawn from the study, the participant will be withdrawn. Study subjects also may elect to withdraw from the study. Patients who withdraw will be asked for their reasons defined as: acupuncture treatment inconvenience; belief that treatment is ineffective; data collection inconvenience; medical reasons; or other. Patients who discontinue participation in the study will be asked if they are willing to provide follow-up data or whether they wish to withdraw completely.

## 14.0 BIOSTATISTICS

The main objective of this study is to provide preliminary data on the effect of acupuncture in reducing persistent CIPN symptoms from baseline to after 8 weeks of acupuncture or sham acupuncture as compared to a usual care control in order to design a fully powered randomized trial of acupuncture versus sham acupuncture. The enrollment goal is 75 patients.

To help plan the definitive trial, we will derive estimates for the standard deviation of all endpoints and the correlation between baseline and follow-up for each endpoint. For each of the patient reported outcomes (NPS, FACT/GOG-NTx, NRS) analysis will be by ANCOVA with the baseline score and most bothersome CIPN symptom as covariates.. Treatment will be coded as dummy variables for any treatment and real acupuncture, hence the three groups will be coded 1/1, 0/1, and 0/0 for acupuncture, Sham acupuncture, and UC respectively. Similarly, to account for type of most bothersome CIPN symptom (tingling vs numbness vs pain) dummy variables for tingling, numbness, and painwill be coded 1/0, 0/1, and 0/0, respectively. Where the week of follow-up is given in the subscript, we will calculate: $NRS_8=\beta_1\ NRS_0 + \beta_2$ Real Acupuncture $+ \beta_3$ Any Acupuncture $+ \beta_4$ Tingling $+ \beta_5$ Numbness + c. The primary endpoint will be NRS of the most bothersome CIPN symptom at 8 weeks.

We will estimate the change in the proportion of patients with a CIPN severity of grade 3 or higher according to the NCI-CTC 4.0 from baseline to 8 and 12 weeks along with an exact 95% confidence interval, separately. To explore the relationship between quantitative sensory testing (QST) and

neuropathy we will use linear regression to assess the association between NRS and TT, VT, and THT at baseline, and the association between change in NRS with change in TT, VT, and THT. For the mechanistic studies, we will use an interaction term between baseline CPM and real acupuncture to predict NRS at 8 weeks: $NRS_8 = \beta_1\ NRS_0 + \beta_2\ CPM_0 + \beta_3$ Real Acupuncture $+ \beta_4\ CPM_0 \times$ Real Acupuncture + c. We will also test acupuncture-induced changes in CPM as $CPM_8 = \beta_1\ CPM_0 + \beta_2$ Real Acupuncture $+ \beta_3$ Any Acupuncture + c. We will also explore the relationship between treatment expectancy and response adjusting for baseline CIPN and treatment arm using multivariable linear regression comparing acupuncture versus sham acupuncture: $NRS_8 = \beta_1\ NRS_0 + \beta_2$ Real Acupuncture $+ \beta_3$ Treatment Expectancy + c. We will also estimate the change in mean HADS, BFI and ISI scores from baseline to 8 and 12 weeks along with a 95% confidence intervals, separately. We will also estimate the proportion of patients with a dose reduction and the proportion of patients who experience a dose escalation of neuropathy medication during the course of the 12 weeks along with an upper 90% confidence interval. All analyses will be by intent to treat, with participants analyzed according to allocation irrespective of treatment actually received. We will use multiple imputation, including 4 week outcome, for missing data at 8 weeks[63]. Patients with 4 and 8 week outcomes are missing, they will not be included in the analysis.

For a trial with 25 patients per group, and an anticipated correlation between baseline and follow-up measures of 0.5, we will be able to calculate the upper bound of a one-sided, 90% confidence interval for the coefficients of any treatment and real acupuncture to approximately 0.35 standard deviations.

Last year, 935 solid tumor survivors had a diagnosis of persistent CIPN 3 months after chemotherapy completion. A recent study showed that among breast cancer survivors 28% reported moderate to severe CIPN 3 months after chemotherapy completion. Therefore, we anticipate 262 patients are eligible per year for our proposed study. Assuming at least a 15% enrollment rate of the eligible patients the estimated accrual is 3-4 patients per month, the estimated accrual period is between 19 and 25 months in order to identify 75 eligible patients.

If the confidence interval for acupuncture treatment effect includes clinically relevant effects, we will then perform a sample size calculation of a future two arm randomized trial using estimates of standard deviation of the CIPN score, the correlation between baseline and followup measures from the current trial. We will calculate the accrual rate along with a 95% Poisson confidence interval (with an exact 95% confidence interval). With a sample size of 75 patients the 95% confidence interval for the accrual rate will be ± 23%. Combining the resulting sample size with the accrual rate will provide an estimate of trial duration, along with an upper and lower bound.

## 15.0 RESEARCH PARTICIPANT REGISTRATION AND RANDOMIZATION PROCEDURES

### 15.1 Research Participant Registration

Confirm eligibility as defined in the section entitled Criteria for Patient/Subject Eligibility.

Obtain informed consent, by following procedures defined in section entitled Informed Consent Procedures.

During the registration process registering individuals will be required to complete a protocol specific Eligibility Checklist.

All participants must be registered through the Protocol Participant Registration (PPR) Office at Memorial Sloan Kettering Cancer Center. PPR is available Monday through Friday from 8:30am – 5:30pm at 646-735-8000. Registrations must be submitted via the PPR Electronic Registration System (http://ppr/). The completed signature page of the written consent/RA or verbal script/RA, a completed Eligibility Checklist and other relevant documents must be uploaded via the PPR Electronic Registration System.

### 15.2 Randomization

Randomization is implemented via MSKCC's Clinical Research Database (CRDB), a secure system that ensures that treatment allocation cannot be guessed before or modified after a patient is registered on trial, allowing for full allocation concealment. Randomization will be 1:1:1 (ACU:Sham: UC) using randomly permuted blocks of random length stratified by type of the most bothersome CIPN symptom (tingling vs numbness vs pain) and severity of such symptom (moderate 4-6 vs 7-10 on NRS). Once the patient is randomized, the CRS or CRM will inform the acupuncturist of the patient's group assignment and the RSA of whether the patient was assigned to an acupuncture group or to the UC group. The RSA's and patients will be blinded to which acupuncture group the patient was assigned. The acupuncturists will be the only ones unblinded. The rest of the study co-investigators and staff will remain blinded.

## 16.0 DATA MANAGEMENT ISSUES

The primary Research Study Assistant (RSA) assigned to this study will be responsible for project compliance, data collection, abstraction and entry, data reporting, regulatory and quality control monitoring, problem identification and prioritization. Coordination of study team activities will be the responsibility of our Research Supervisor and/or Research Manager.

All data and forms gathered for this study will be collected and stored in a secure location in the facilities of the Integrative Medicine Service. The data will be entered into either CRDB, Excel, REDCap, or Access secure databases based on the database functionality. Access will be used to store the scannable patient-reported outcomes data associated with the AutoData Scannable Office software described below. Excel will be used as a study tracker, for screening information and for the QST/CPM data, and CRDB will be used to capture minimal dataset and other study data. Participants will be asked to complete patient reported outcomes assessments online using REDCap, as described below. If they prefer, patients will have the option to complete the measures via pencil and paper on scannable forms or over the phone with an RSA to reduce participant burden and ensure timely completion.

REDCap (Research Electronic Data Capture) is a data management software system supported by the Clinical Research Administration (CRA) at MSK. Members of the CRA supporting the REDCap software will have access to REDCap projects hosted by MSK's servers for the purpose of ensuring the proper functioning of the database and the overall software system. REDCap is a tool for the creation of customized, secure data management systems including web-based data entry forms, reporting tools, and a full array of security features including user- and group-based privileges with a full audit trail of data manipulation an export procedures. REDCap is maintained on MSK-owned servers that are kept in a locked server room with appropriate environmental modifications (e.g. proper ventilation, power redundancy and fault tolerance arrangement) and backed up nightly with some back-up tapes stored off-site. The MSK Information Systems group is responsible for applying all operating system patches and security updates to the REDCap servers. All connections to REDCap utilize encrypted (SSL-based) connections. Nationally, the REDCap software is developed, enhanced, and supported through a multi-institutional consortium led by Vanderbilt University.

The questionnaires that patients complete for this protocol will not be IRB stamped documents. The questionnaires will not be changed from their IRB stamped counterparts, but in order to use these questionnaires, we need to have them in Microsoft Word format. The software (AutoData Scannable Office) we will used takes these Microsoft Word documents and prints them with a patient specific barcode at the bottom. After the patient fills it out, the original questionnaire can be scanned into this software and the software reads and records the patients' answers into a Microsoft Access database, where the patient-reported data will be stored. We have successfully utilized this process and it significantly reduces data entry time and errors.

Source documentation will be available to support the computerized patient data. The confidentiality of patient information will be carefully protected. Following data entry by Integrative Medicine Service research staff, data will be maintained in a secure location in the Integrative Medicine offices. All data will be stored in a fashion consistent with FDA guidelines (21CFR11 compliant) and HIPAA security rules.

### 16.1 Quality Assurance

Regular registration reports will be generated to monitor patient accruals and completeness of registration data. Routine data quality reports will be generated to assess missing data and inconsistencies. Accrual rates and extent and accuracy of evaluations and follow-up will be monitored periodically throughout the study period and potential problems will be brought to the attention of the study team for discussion and action. Random-sample data quality and protocol compliance audits will be conducted by the study team, at a minimum of two times per year, more frequently if indicated.

### 16.2 Data and Safety Monitoring

The Data and Safety Monitoring (DSM) Plans at Memorial Sloan-Kettering Cancer Center were approved by the National Cancer Institute in September 2001. The plans address the new policies set forth by the NCI in the document entitled "Policy of the National Cancer Institute for Data and Safety Monitoring of Clinical Trials" which can be found at: http://cancertrials.nci.nih.gov/researchers/dsm/index.html. The DSM Plans at MSK were established and are monitored by the Office of Clinical Research. The MSK Data and Safety Monitoring Plans can be found on the MSK Intranet at: *http://mskweb2.mskcc.orglirb/index.htm*

There are several different mechanisms by which clinical trials are monitored for data, safety and quality. There are institutional processes in place for quality assurance (e.g., protocol monitoring, compliance and data verification audits, therapeutic response, and staff education on clinical research QA) and departmental procedures for quality control, plus there are two institutional committees that are responsible for monitoring the activities of our clinical trials programs. The committees: *Data and Safety Monitoring Committee (OSMC)* for Phase I and II clinical trials, and the *Data and Safety Monitoring Board (OSMB)* for Phase III clinical trials, report to the Center's Research Council and Institutional Review Board.

During the protocol development and review process, each protocol will be assessed for its level of risk and degree of monitoring required. Every type of protocol (e.g., NIH sponsored, in-house sponsored, industrial sponsored, NCI cooperative group, etc.) will be addressed and the monitoring procedures will be established at the time of protocol activation.

## 17.0 PROTECTION OF HUMAN SUBJECTS

Benefits: Study subjects may have reduced CIPN severity.

Risks: Reports of serious adverse events from acupuncture in the research literature are rare. In the more than ten-year experience of Integrative Medicine acupuncture treatment, we have never had a related serious AE.

Side-effects: It is conceivable that a patient may feel some discomfort from the acupuncture needles, or that the needles may cause a minor bruise. However, these would likely be very minor, as acupuncture needles are filiform and hair-thin, much smaller than standard hypodermic needles. They are also sterile, single-use and applied following alcohol swab. Standard of care procedures will be followed to minimize risk.There may be other unforeseeable risks.

Consent Process: Participation in this trial is voluntary. All study subjects will be required to sign a statement of informed consent, which will conform to local IRB guidelines. Participation in this study will take place only after signed informed consent is obtained.

Costs: Study subjects will not be charged for acupuncture received in this study nor will they be charged for quantitative sensory testing. Subjects will not be paid for their participation.

Confidentiality: Every effort will be made to maintain confidentiality of the study subjects. Research and hospital records are confidential. Subject's names or any other personally identifying information will not be used in reports or publications resulting from this study. Authorized agencies (e.g., qualified monitors from NCI etc.), and appropriate personnel may review subject's records as

required. All forms are kept in a locked file cabinet when not in use. Clinical data will be kept in centralized databases with restricted access to study personnel. Data will be entered into MSK's clinical research database (CRDB) a study excel file, and study Access and REDCap databases on a secure MSK shared drive. Individual identifying information will be omitted from figures used in publications resulting from this research. No genetic tests will be performed in this study. Data will be reported in the aggregate.

Risk of psychological distress: Some of the questions in the questionnaire may elicit distress for some patients. If a patient demonstrates clinically significant distress, s/he will be referred to the appropriate clinical/psychosocial services. An appropriate referral will be made if anxiety (7 items) or depression (7 items) scores for the HADS is greater than 10 as 11-21 indicates clinically significant depression/anxiety.

### 17.1 Privacy

MSK's Privacy Office may allow the use and disclosure of protected health information pursuant to a completed and signed Research Authorization form. The use and disclosure of protected health information will be limited to the individuals described in the Research Authorization form. A Research Authorization form must be completed by the Principal Investigator and approved by the IRB and Privacy Board (IRB/PB).

### 17.2 Serious Adverse Event (SAE) Reporting

An adverse event is considered serious if it results in ANY of the following outcomes:

- Death
- A life-threatening adverse event
- An adverse event that results in inpatient hospitalization or prolongation of existing hospitalization
- A persistent or significant incapacity or substantial disruption of the ability to conduct normal life functions
- A congenital anomaly/birth defect
- Important Medical Events (IME) that may not result in death, be life threatening, or require hospitalization may be considered serious when, based upon medical judgment, they may jeopardize the patient or subject and may require medical or surgical intervention to prevent one of the outcomes listed in this definition

Note: Hospital admission for a planned procedure/disease treatment is not considered an SAE.

SAE reporting is required as soon as the participant signs consent. SAE reporting is required for 30-days after the participant's last investigational treatment or intervention. Any events that occur after the 30-day period and that are at least possibly related to protocol treatment must be reported.

If an SAE requires submission to the IRB office per IRB SOP RR-408 'Reporting of Serious Adverse Events', the SAE report must be sent to the IRB within 5 calendar days of the event. SAE's classified as unrelated to the study intervention (per section 11.0) will not be

considered reportable to the IRB and will not have a corresponding Clinical Research Database (CRDB) SAE report generated). The IRB requires a Clinical Research Database (CRDB) SAE report be submitted electronically to the SAE Office as follows:

For IND/IDE trials: Reports that include a Grade 5 SAE should be sent to saegrade5@mskcc.org. All other reports should be sent to saemskind@mskcc.org.

For all other trials: Reports that include a Grade 5 SAE should be sent to saegrade5@mskcc.org. All other reports should be sent to sae@mskcc.org.

The report should contain the following information:

Fields populated from CRDB:

- Subject's initials
- Medical record number
- Disease/histology (if applicable)
- Protocol number and title

Data needing to be entered:

- The date the adverse event occurred
- The adverse event
- The grade of the event
- Relationship of the adverse event to the treatment (drug, device, or intervention)
- If the AE was expected
- The severity of the AE
- The intervention
- Detailed text that includes the following
    - A explanation of how the AE was handled
    - A description of the subject's condition
    - Indication if the subject remains on the study
- If an amendment will need to be made to the protocol and/or consent form
- If the SAE is an Unanticipated Problem

The PI's signature and the date it was signed are required on the completed report.

For IND/IDE protocols:
The CRDB SAE report should be completed as per above instructions. If appropriate, the report will be forwarded to the FDA by the SAE staff through the IND Office

**17.2.1 N/A**

## 18.0 INFORMED CONSENT PROCEDURES

Before protocol-specified procedures are carried out, consenting professionals will explain full details of the protocol and study procedures as well as the risks involved to participants prior to their inclusion in the study. Participants will also be informed that they are free to withdraw from the study at any time. All participants must sign an IRB/PB-approved consent form indicating their consent to participate. This consent form meets the requirements of the Code of Federal Regulations and the Institutional Review Board/Privacy Board of this Center. The consent form will include the following:

1. The nature and objectives, potential risks and benefits of the intended study.
2. The length of study and the likely follow-up required.
3. Alternatives to the proposed study. (This will include available standard and investigational therapies. In addition, patients will be offered an option of supportive care for therapeutic studies.)
4. The name of the investigator(s) responsible for the protocol.
5. The right of the participant to accept or refuse study interventions/interactions and to withdraw from participation at any time.

Before any protocol-specific procedures can be carried out, the consenting professional will fully explain the aspects of patient privacy concerning research specific information. In addition to signing the IRB Informed Consent, all patients must agree to the Research Authorization component of the informed consent form.

Each participant and consenting professional will sign the consent form. The participant must receive a copy of the signed informed consent form.

## 19.0 REFERENCES

1. American Cancer Society: Cancer Facts & Figures 2016,
2. Schneider BP, Hershman DL, Loprinzi C: Symptoms: Chemotherapy-Induced Peripheral Neuropathy. Adv Exp Med Biol 862:77-87, 2015
3. Ewertz M, Qvortrup C, Eckhoff L: Chemotherapy-induced peripheral neuropathy in patients treated with taxanes and platinum derivatives. Acta Oncol 54:587-91, 2015
4. Seretny M, Currie GL, Sena ES, et al: Incidence, prevalence, and predictors of chemotherapy-induced peripheral neuropathy: A systematic review and meta-analysis. Pain 155:2461-70, 2014
5. Bao T, Basal C, Seluzicki C, et al: Long-term chemotherapy-induced peripheral neuropathy among breast cancer survivors: prevalence, risk factors, and fall risk. Breast Cancer Res Treat 159:327-33, 2016
6. Jongen JL, Broijl A, Sonneveld P: Chemotherapy-induced peripheral neuropathies in hematological malignancies. J Neurooncol 121:229-37, 2015
7. Pachman DR, Barton DL, Watson JC, et al: Chemotherapy-induced peripheral neuropathy: prevention and treatment. Clinical Pharmacology and Therapeutics 90:377-87, 2011
8. Mols F, Beijers T, Lemmens V, et al: Chemotherapy-Induced Neuropathy and Its Association With Quality of Life Among 2- to 11-Year Colorectal Cancer Survivors: Results From the Population-Based PROFILES Registry. Journal of Clinical Oncology, 2013

9. Jensen MP, Friedman M, Bonzo D, et al: The validity of the neuropathic pain scale for assessing diabetic neuropathic pain in a clinical trial. Clinical Journal of Pain 22:97-103, 2006

10. Hammack JE, Michalak JC, Loprinzi CL, et al: Phase III evaluation of nortriptyline for alleviation of symptoms of cis-platinum-induced peripheral neuropathy. Pain 98:195-203, 2002

11. Rao RD, Michalak JC, Sloan JA, et al: Efficacy of gabapentin in the management of chemotherapy-induced peripheral neuropathy: a phase 3 randomized, double-blind, placebo-controlled, crossover trial (N00C3). Cancer 110:2110-8, 2007

12. Rao RD, Flynn PJ, Sloan JA, et al: Efficacy of lamotrigine in the management of chemotherapy-induced peripheral neuropathy: a phase 3 randomized, double-blind, placebo-controlled trial, N01C3. Cancer 112:2802-8, 2008

13. Smith EM, Pang H, Cirrincione C, et al: Effect of duloxetine on pain, function, and quality of life among patients with chemotherapy-induced painful peripheral neuropathy: a randomized clinical trial. JAMA : the journal of the American Medical Association 309:1359-67, 2013

14. Hershman DL, Lacchetti C, Dworkin RH, et al: Prevention and management of chemotherapy-induced peripheral neuropathy in survivors of adult cancers: American Society of Clinical Oncology clinical practice guideline. Journal of clinical oncology : official journal of the American Society of Clinical Oncology 32:1941-67, 2014

15. Cymbalta Approval History http://www.drugs.com/history/cymbalta.html, 2014

16. Abuaisha BB, Costanzi JB, Boulton AJ: Acupuncture for the treatment of chronic painful peripheral diabetic neuropathy: a long-term study. Diabetes Research and Clinical Practice 39:115-21, 1998

17. Ahn AC, Bennani T, Freeman R, et al: Two styles of acupuncture for treating painful diabetic neuropathy--a pilot randomised control trial. Acupuncture in Medicine 25:11-7, 2007

18. Galantino ML, Eke-Okoro ST, Findley TW, et al: Use of noninvasive electroacupuncture for the treatment of HIV-related peripheral neuropathy: a pilot study. Journal of Alternative and Complementary Medicine 5:135-42, 1999

19. Bao T, Goloubeva O, Pelser C, et al: A pilot study of acupuncture in treating bortezomib-induced peripheral neuropathy in patients with multiple myeloma. Integrative Cancer Therapies 13:396-404, 2014

20. Garcia MK, Cohen L, Guo Y, et al: Electroacupuncture for thalidomide/bortezomib-induced peripheral neuropathy in multiple myeloma: a feasibility study. Journal of hematology & oncology 7:41, 2014

21. Rosenberg NR, Portegies P, de Visser M, et al: Diagnostic investigation of patients with chronic polyneuropathy: evaluation of a clinical guideline. J Neurol Neurosurg Psychiatry 71:205-9, 2001

22. Wang WS, Lin JK, Lin TC, et al: Oral glutamine is effective for preventing oxaliplatin-induced neuropathy in colorectal cancer patients. Oncologist 12:312-9, 2007

23. Backonja MM, Attal N, Baron R, et al: Value of quantitative sensory testing in neurological and pain disorders: NeuPSIG consensus. Pain 154:1807-19, 2013

24. Krumova EK, Geber C, Westermann A, et al: Neuropathic pain: is quantitative sensory testing helpful? Curr Diab Rep 12:393-402, 2012

25. Hershman DL, Weimer LH, Wang A, et al: Association between patient reported outcomes and quantitative sensory tests for measuring long-term neurotoxicity in breast cancer survivors treated with adjuvant paclitaxel chemotherapy. Breast Cancer Res Treat 125:767-74, 2011

26. Takeshige C, Sato T, Mera T, et al: Descending pain inhibitory system involved in acupuncture analgesia. Brain Res Bull 29:617-34, 1992

27. Liu J, Fu W, Yi W, et al: Extrasegmental analgesia of heterotopic electroacupuncture stimulation on visceral pain rats. Brain Res 1373:160-71, 2011

28. Zhao ZQ: Neural mechanism underlying acupuncture analgesia. Progress in neurobiology 85:355-75, 2008

29. Pud D, Granovsky Y, Yarnitsky D: The methodology of experimentally induced diffuse noxious inhibitory control (DNIC)-like effect in humans. Pain 144:16-9, 2009
30. Yarnitsky D, Arendt-Nielsen L, Bouhassira D, et al: Recommendations on terminology and practice of psychophysical DNIC testing. Eur J Pain 14:339, 2010
31. Pielsticker A, Haag G, Zaudig M, et al: Impairment of pain inhibition in chronic tension-type headache. Pain 118:215-23, 2005
32. Julien N, Goffaux P, Arsenault P, et al: Widespread pain in fibromyalgia is related to a deficit of endogenous pain inhibition. Pain 114:295-302, 2005
33. Sandrini G, Rossi P, Milanov I, et al: Abnormal modulatory influence of diffuse noxious inhibitory controls in migraine and chronic tension-type headache patients. Cephalalgia 26:782-9, 2006
34. Maixner W, Fillingim R, Booker D, et al: Sensitivity of patients with painful temporomandibular disorders to experimentally evoked pain. Pain 63:341-51, 1995
35. Song GH, Venkatraman V, Ho KY, et al: Cortical effects of anticipation and endogenous modulation of visceral pain assessed by functional brain MRI in irritable bowel syndrome patients and healthy controls. Pain 126:79-90, 2006
36. Yarnitsky D, Granot M, Nahman-Averbuch H, et al: Conditioned pain modulation predicts duloxetine efficacy in painful diabetic neuropathy. Pain 153:1193-8, 2012
37. Vincent C: The safety of acupuncture. BMJ 323:467-8, 2001
38. Alimi D, Rubino C, Pichard-Leandri E, et al: Analgesic effect of auricular acupuncture for cancer pain: a randomized, blinded, controlled trial. Journal of Clinical Oncology 21:4120-6, 2003
39. Lao L, Bergman S, Hamilton GR, et al: Evaluation of acupuncture for pain control after oral surgery: a placebo-controlled trial. Archives of otolaryngology--head & neck surgery 125:567-72, 1999
40. Berman BM, Lao L, Langenberg P, et al: Effectiveness of acupuncture as adjunctive therapy in osteoarthritis of the knee: a randomized, controlled trial. Annals of internal medicine 141:901-10, 2004
41. Childs JD, Piva SR, Fritz JM: Responsiveness of the numeric pain rating scale in patients with low back pain. Spine (Phila Pa 1976) 30:1331-4, 2005
42. Farrar JT, Young JP, Jr., LaMoreaux L, et al: Clinical importance of changes in chronic pain intensity measured on an 11-point numerical pain rating scale. Pain 94:149-58, 2001
43. Downie WW, Leatham PA, Rhind VM, et al: Studies with pain rating scales. Ann Rheum Dis 37:378-81, 1978
44. Ferraz MB, Quaresma MR, Aquino LR, et al: Reliability of pain scales in the assessment of literate and illiterate patients with rheumatoid arthritis. J Rheumatol 17:1022-4, 1990
45. Calhoun EA, Welshman EE, Chang CH, et al: Psychometric evaluation of the Functional Assessment of Cancer Therapy/Gynecologic Oncology Group-Neurotoxicity (Fact/GOG-Ntx) questionnaire for patients receiving systemic chemotherapy. Int J Gynecol Cancer 13:741-8, 2003
46. Galer BS, Jensen MP: Development and preliminary validation of a pain measure specific to neuropathic pain: the Neuropathic Pain Scale. Neurology 48:332-8, 1997
47. Jensen MP, Friedman M, Bonzo D, et al: The validity of the neuropathic pain scale for assessing diabetic neuropathic pain in a clinical trial. Clin J Pain 22:97-103, 2006
48. Fishbain DA, Lewis JE, Cutler R, et al: Can the neuropathic pain scale discriminate between non-neuropathic and neuropathic pain? Pain Med 9:149-60, 2008
49. I. K: Changing expectaions: A key to effective psychotherapy (ed First). Pacific Grove, CA: Brooks, Cole Publishing Company, 1990
50. Colagiuri B, Smith CA: A systematic review of the effect of expectancy on treatment responses to acupuncture. Evid Based Complement Alternat Med 2012:857804, 2012
51. Mao JJ, Armstrong K, Farrar JT, et al: Acupuncture expectancy scale: development and preliminary validation in China. Explore (NY) 3:372-7, 2007

52. Mao JJ, Xie SX, Bowman MA: Uncovering the expectancy effect: the validation of the acupuncture expectancy scale. Altern Ther Health Med 16:22-7, 2010

53. Bauml J, Xie SX, Farrar JT, et al: Expectancy in real and sham electroacupuncture: does believing make it so? J Natl Cancer Inst Monogr 2014:302-7, 2014

54. Geber C, Klein T, Azad S, et al: Test-retest and interobserver reliability of quantitative sensory testing according to the protocol of the German Research Network on Neuropathic Pain (DFNS): a multi-centre study. Pain 152:548-56, 2011

55. Drobek W, De Laat A, Schoenaers J: Tactile threshold and pressure pain threshold during treatment of orofacial pain: an explorative study. Clin Oral Investig 5:185-93, 2001

56. Goldberg JM, Lindblom U: Standardised method of determining vibratory perception thresholds for diagnosis and screening in neurological investigation. J Neurol Neurosurg Psychiatry 42:793-803, 1979

57. Yarnitsky D: Quantitative sensory testing. Muscle Nerve 20:198-204, 1997

58. Granot M, Weissman-Fogel I, Crispel Y, et al: Determinants of endogenous analgesia magnitude in a diffuse noxious inhibitory control (DNIC) paradigm: do conditioning stimulus painfulness, gender and personality variables matter? Pain 136:142-9, 2008

59. Morin CM, Belleville G, Belanger L, et al: The Insomnia Severity Index: psychometric indicators to detect insomnia cases and evaluate treatment response. Sleep 34:601-8, 2011

60. Savard MH, Savard J, Simard S, et al: Empirical validation of the Insomnia Severity Index in cancer patients. Psychooncology 14:429-41, 2005

61. Bjelland I, Dahl AA, Haug TT, et al: The validity of the Hospital Anxiety and Depression Scale. An updated literature review. J Psychosom Res 52:69-77, 2002

62. Mendoza TR, Wang XS, Cleeland CS, et al: The rapid assessment of fatigue severity in cancer patients: use of the Brief Fatigue Inventory. Cancer 85:1186-96, 1999

63. Vickers AJ, Altman DG: Statistics notes: missing outcomes in randomised trials. BMJ 346:f3438, 2013

64. wikipedia: Activities of daily living, 2016

## 20.0 20.0 APPENDICES

- **Appendix A: Patient Reported Outcomes – Scannable Forms**
    - Numeric Rating Scale
    - Neuropathy Pain Scale
    - Treatment Expectancy Scale
    - FACT/GOG-Ntx questionnaire
    - Brief Fatigue Inventory
    - Insomnia Severity Index
    - Hospital Anxiety and Depression Scale

**Appendix B: Severity of CIPN as defined by NCI-CTC 4.0**
**Appendix C: ECOG performance Status Scale**
**Appendix D: Pain Medication Log**
**Appendix E: Recruitment Letter**
**Appendix F: Recruitment Flyers**
**Appendix G: Study Allocation Belief**
