# Supplementary material for: Effect of Acupuncture vs Sham Procedure on Chemotherapy-Induced Peripheral Neuropathy Symptoms: A Randomized Clinical Trial
Source: JAMA Netw Open. 2020 Mar 11;3(3):e200681. doi: 10.1001/jamanetworkopen.2020.0681 (PMC7066475; doi:10.1001/jamanetworkopen.2020.0681)
Supplement: Supplement 2. — Data Sharing Statement [file jamanetwopen-3-e200681-s002.pdf]

Data Sharing Statement

Bao T, Patil S, Chen C, et al. Effect of acupuncture vs sham procedure on chemotherapy-induced peripheral neuropathy symptoms: a randomized clinical trial. *JAMA Netw Open*. 2020;3(3):e200681. doi:10.1001/jamanetworkopen.2020.0681

Data

Data available: Yes
Data types: Deidentified participant data
How to access data: baot@mskcc.org
When available: With publication

Supporting Documents

Document types: Informed consent form
How to access documents: baot@mskcc.org
When available: With publication

Additional Information

Who can access the data: researchers whose proposed use of the data has been approved
Types of analyses: for research
Mechanisms of data availability: after approval of a proposal and with a signed data access agreement
Any additional restrictions: none
